# Genome-wide identification of the longan R2R3-MYB gene family and its role in primary and lateral root

**DOI:** 10.1186/s12870-023-04464-9

**Published:** 2023-09-23

**Authors:** Xinmin Lv, Shichang Tian, Shilian Huang, Junbin Wei, Dongmei Han, Jianguang Li, Dongliang Guo, Yan Zhou

**Affiliations:** 1 Key Laboratory of South Subtropical Fruit Biology and Genetic Resource Utilization, Ministry of Agriculture, Institute of Fruit Tree Research, Guangdong Academy of Agricultural Sciences, Guangzhou, 510640 China; 2 Life Science and Technology School, Lingnan Normal University, Zhanjiang, 524048 China

**Keywords:** Longan, R2R3-MYB, miRNAs, Root development

## Abstract

R2R3-MYB is an important transcription factor family that regulates plant growth and development. Root development directly affects the absorption of water and nutrients by plants. Therefore, to understand the regulatory role of R2R3-MYB transcription factor family in root development of longan, this study identified the R2R3-MYB gene family members at the genome-wide level, and analyzed their phylogenetic characteristics, physical and chemical properties, gene structure, chromosome location and tissue expression. The analysis identified 124 R2R3-MYB family members in the longan genome. Phylogenetic analysis divided these members into 22 subfamilies, and the members of the unified subfamily had similar motifs and gene structures. The result of qRT-PCR showed that expression levels of *DlMYB33*, *DlMYB34*, *DlMYB59*, and *DlMYB77* were significantly higher in main roots than in lateral as opposed to those of *DlMYB35*, *DlMYB69*, *DlMYB70*, and *DlMYB83,* which were significantly lower. SapBase database prediction and miRNAs sequencing results showed that 34 longan miRNAs could cleave R2R3-MYB, including 17 novel miRNAs unique to longan. The qRT-PCR and subcellular localization experiments of DlMYB92 and DlMYB98 showed that DlMYB92 is a key factor that regulates transcription in the nucleus and participates in the regulation of longan lateral root development. Longan also has a conserved miRNA-MYB-lateral root development regulation mechanism. This study provides a reference for further research on the transcriptional regulation of the miRNA-R2R3-MYB module in the root development of longan.

**Supplementary Information:**

The online version contains supplementary material available at 10.1186/s12870-023-04464-9.

## Introduction

Gene expression in plants is regulated by several factors at the transcriptional and post-transcriptional levels. Transcription factors (TFs) are a class of trans-acting factors that regulate gene expression at the transcriptional level [1, 2]. The MYB (v-Myb avian myeloblastosis viral oncogene homolog) transcription factor family is a large and functionally diverse family of TFs unique to plants [3], and the TFs are classified into four types based on the number of structural domains in the amino acid sequence: 1R-MYB, R2R3-MYB, 3R-MYB, and 4R-MYB. R2R3-MYB contains two tandem MYB structural domains, R2 and R3. [4]. MicroRNAs (miRNAs), a class of single-stranded endogenous non-coding RNAs ranging in size from 20 ~ 24 nt, play an important role as post-transcriptional regulators [5]. Recent research has shown that miRNAs play a critical role in plant growth and resistance to stress, and have become key molecules in understanding the molecular basis of biological activity [6–9]. Studies show that miRNAs exert their regulatory roles by targeting TFs [10, 11]. Therefore, understanding MYB regulation of target genes at the transcriptional level and miRNA regulation of MYB at the post-transcriptional level is important for insights into plant growth and development.

Root growth and development directly affect plant uptake of minerals and water [12]. While plant roots exhibit high phenotypic plasticity [13], their growth and development are regulated by multiple factors. It has been found that miRNAs and TFs can form miRNA-TFs regulatory modules to regulate plant root development in a stepwise manner. For example, MYB interacts with abscisic acid (ABA) signaling in the endodermis of *Arabidopsis* roots to form a cascading regulatory network of lignification and corkiness that affects lateral root development [14]. In *Arabidopsis AtmmiR159ab* double mutants, the expression of *MYB33*, *MYB65*, and *MYB101* increased, the primary root became significantly longer, and the meristematic tissue became larger. The number of cells in the meristematic and elongation zones increased significantly compared with the wild type, indicating that miR159 may inhibit primary root growth by cleaving *MYB33*, *MYB65*, and *MYB101* [15]. LAC2 (LACCASE2), a negative regulator of lignin deposition in root vascular tissue, is post-transcriptionally regulated by miR397b under water-deficit conditions, resulting in increased root length and reduced lignin content in *Arabidopsis* root vascular tissue [16]. Additionally, It was also demonstrated that *Arabidopsis* miR156 represses adventitious root development by regulating the target gene SPL (SQUAMOSA promoter binding protein-like) [17]. The miR156-SPL regulatory module was found to play a role in root development in rice [18], maize [19], alfalfa [20], and apple [21]. In summary, miRNA and MYB TFs are important regulators of plant root development. However, the role of most miRNAs in regulating root development has not been directly validated in transgenic plants due to the limitations of transgenic technology.

Longan (*Dimocarpus longan*) is widely cultivated in many Southeast Asian countries, including China [22, 23]. Despite the large cultivation area of longan in China, yields are low and still need improvement [24]. Irrational fertilization of land during cultivation has been shown to cause the degradation of longan roots, resulting in weaker root nutrient uptake and limiting yield improvement. In order to effectively enhance the quantity of lateral roots and their accompanying nutrient absorption capacity, a comprehensive understanding of how miRNA and MYB regulate the development of longan roots is imperative. Consequently, members of the R2R3-MYB transcription factor family in longan were screened and identified through whole genome sequencing. MicroRNA sequencing was used to analyze the post-transcriptional regulation of the R2R3-MYB family members in longan [25, 26]. This work will facilitate our further understanding of the potential of MYB TFs and miRNAs in regulating root development in longan.

## Results

### Identification and physicochemical characterization of the R2R3-MYB gene family in longan

The identification of R2R3-MYB gene family members in longan was carried out through an HMM search and Batch-CDD-search, targeting protein sequences with the R2R3-MYB structural domain. A total of 124 R2R3-MYB gene family members were successfully identified in the ‘Shi Xia’ longan genome and designated as DlMYB1-DlMYB124 (Table 1). The lengths of the protein sequences of the124 longan R2R3-MYB family members ranged from 134 to 661 aa, with an average length of 307 aa. The molecular masses ranged from 15.69 to 775.81 kDa, with an average molecular mass of 34.68 kDa. Theoretical isoelectric points ranged from 4.91 to 10.6. The instability coefficients ranged from 35.62 to 72.31, and only DlMYB101, DlMYB21, DlMYB91, DlMYB58, DlMYB119, DlMYB20 and DlMYB114 proteins had instability coefficients less than 40, while the rest of the 117 R2R3-MYB proteins had instability coefficients greater than 40, indicating that these proteins were unstable. The fat coefficients ranged from 50.78 to 84.16, indicating that the proteins encoded by the R2R3-MYB transcription factor gene of D. longan were thermally stable. The average coefficients of hydrophilicity ranged from -1.22 to -0.5, meaning that all longan R2R3-MYBs were hydrophilic proteins.

**Table 1:** Information of *Dimocarpus longan* R2R3-MYB gene family

**Gene Name**	**GeneID**	**Protein length (aa)**	**Molecular weight (Da)**	**Theoretical isoelectric point**	**Instability index**	**Aliphatic index**	**GRAVY**
*DlMYB1*	Dil.01g001450.1.t1	199	23176	10.47	51.11	77.44	-0.723
*DlMYB2*	Dil.01g001460.1.t1	304	34707.75	5.55	47.64	70.86	-0.813
*DlMYB3*	Dil.01g001470.1.t1	284	32235.32	6.33	53.76	78.7	-0.691
*DlMYB4*	Dil.01g003150.1.t1	205	23835.94	9.18	52.02	62.34	-1.018
*DlMYB5*	Dil.01g003190.1.t1	259	29542.67	8.93	42.71	77.22	-0.754
*DlMYB6*	Dil.01g003200.1.t1	303	34075.46	5.56	43.73	74.32	-0.622
*DlMYB7*	Dil.01g003210.1.t1	284	32125.82	6	53.36	64.23	-0.856
*DlMYB8*	Dil.01g004190.1.t1	323	35446.5	6.79	47.12	60.43	-0.621
*DlMYB9*	Dil.01g008340.1.t1	311	35128.06	5.35	53.67	67.75	-0.852
*DlMYB10*	Dil.01g011140.1.t1	231	26933.41	8.82	67.18	63.33	-0.922
*DlMYB11*	Dil.01g013170.1.t1	449	50769.17	7.05	43.99	62.54	-0.949
*DlMYB12*	Dil.01g013560.1.t1	376	42118.85	6.26	56.8	68.06	-0.768
*DlMYB13*	Dil.01g027080.1.t1	268	30207.94	5.76	60.29	65.9	-0.679
*DlMYB14*	Dil.01g027880.1.t1	268	31214.1	9.11	65.27	65.93	-0.874
*DlMYB15*	Dil.01g028020.1.t1	268	31214.1	9.11	65.27	65.93	-0.874
*DlMYB16*	Dil.01g035440.1.t1	312	35399.27	6.13	51.25	64.13	-0.7
*DlMYB17*	Dil.02g008180.1.t1	168	19809.19	6.24	52.8	62.08	-0.976
*DlMYB18*	Dil.02g019190.1.t1	340	38216.84	6.61	59.12	81.18	-0.597
*DlMYB19*	Dil.02g022740.1.t1	313	34729.78	9.97	59.13	81.05	-0.558
*DlMYB20*	Dil.02g026310.1.t2	323	35699.19	7.6	36.79	64.71	-0.638
*DlMYB21*	Dil.02g026520.1.t1	323	35715.24	7.6	37.71	65.91	-0.622
*DlMYB22*	Dil.03g001010.1.t1	274	31094.8	6.43	55.97	67.63	-0.672
*DlMYB23*	Dil.03g001500.1.t1	258	29533.21	6.55	49.76	76.36	-0.785
*DlMYB24*	Dil.03g001510.1.t2	191	22382.64	10.6	66.6	80.16	-1.019
*DlMYB25*	Dil.03g001540.1.t1	196	22797.76	8.66	63.48	72.14	-0.993
*DlMYB26*	Dil.03g001770.1.t1	196	22797.76	8.66	63.48	72.14	-0.993
*DlMYB27*	Dil.03g002760.1.t1	413	46101.47	6.23	72.31	72.25	-0.583
*DlMYB28*	Dil.03g024290.1.t1	311	35229.81	5.12	59.29	59.29	-0.752
*DlMYB29*	Dil.04g001260.1.t1	314	35590.88	5.25	65.63	71.08	-0.713
*DlMYB30*	Dil.04g001310.1.t1	313	35447.74	5.33	65.65	71.31	-0.704
*DlMYB31*	Dil.04g003980.1.t1	306	34046.41	6.11	64.38	67.03	-0.638
*DlMYB32*	Dil.04g011620.1.t1	391	43656.37	4.91	47.58	65.6	-0.747
*DlMYB33*	Dil.04g012460.1.t1	307	34731.26	5.77	54.22	66.06	-0.781
*DlMYB34*	Dil.04g013360.1.t1	451	49984.53	6.73	59.58	65.57	-0.665
*DlMYB35*	Dil.04g015690.1.t1	284	32203.09	8.86	43.49	74.47	-0.908
*DlMYB36*	Dil.04g026030.1.t1	289	32370.15	8.76	53.33	67.47	-0.688
*DlMYB37*	Dil.04g027150.1.t1	289	32402.21	8.76	54.19	66.47	-0.696
*DlMYB38*	Dil.04g027320.1.t1	289	32370.15	8.76	53.33	67.47	-0.688
*DlMYB39*	Dil.04g029230.1.t1	268	29718.11	8.66	53.78	61.49	-0.782
*DlMYB40*	Dil.05g000460.1.t1	453	50471.69	6.92	46.01	58.59	-0.807
*DlMYB41*	Dil.05g012930.1.t1	134	15691.01	9.82	42.55	77.91	-0.892
*DlMYB42*	Dil.05g016520.1.t1	350	38953.18	6.8	44.88	65.23	-0.807
*DlMYB43*	Dil.05g016600.1.t1	393	42825.94	9.16	44.67	66.06	-0.697
*DlMYB44*	Dil.05g017290.1.t1	334	36767.88	6.31	40.61	67.46	-0.76
*DlMYB45*	Dil.06g001770.1.t1	179	20793.77	8.94	46	76.82	-0.684
*DlMYB46*	Dil.06g008360.1.t1	358	40204.05	5.75	50.46	69.22	-0.615
*DlMYB47*	Dil.06g011210.1.t1	263	29835.79	9.37	50.61	71.56	-0.757
*DlMYB48*	Dil.06g011270.1.t1	263	29819.83	9.45	51.84	72.66	-0.728
*DlMYB49*	Dil.06g020760.1.t1	344	38036.87	6.55	58.74	78.84	-0.521
*DlMYB50*	Dil.07g009490.1.t1	374	42419.15	5.57	57	69.57	-0.577
*DlMYB51*	Dil.07g017900.1.t1	374	42892.59	5.66	46.48	65.13	-0.812
*DlMYB52*	Dil.07g018010.1.t2	271	31383.98	5.79	50.98	54.72	-0.801
*DlMYB53*	Dil.08g001020.1.t1	173	20081.37	9.78	53.46	74.39	-0.828
*DlMYB54*	Dil.08g001740.1.t1	281	32250.44	7.64	44.09	70.82	-0.733
*DlMYB55*	Dil.08g001750.1.t1	246	28152.3	10.05	43.86	76.95	-0.755
*DlMYB56*	Dil.08g001760.1.t1	282	32307.56	9	40.15	70.89	-0.762
*DlMYB57*	Dil.08g001770.1.t1	661	75808.17	6.33	52.74	80.54	-0.5
*DlMYB58*	Dil.08g002130.1.t1	222	25196.46	7.1	37.33	74.23	-0.735
*DlMYB59*	Dil.08g014150.1.t1	286	32796.13	5.26	50.31	59.34	-0.991
*DlMYB60*	Dil.08g015750.1.t1	298	33591.65	6.27	46.71	65.74	-0.699
*DlMYB61*	Dil.08g015770.1.t1	305	34169.82	5.89	46.88	67.18	-0.735
*DlMYB62*	Dil.08g019300.1.t2	309	35665.16	6.1	52.89	51.13	-1.22
*DlMYB63*	Dil.08g020010.1.t1	353	39857.74	6.21	60.09	70.96	-0.599
*DlMYB64*	Dil.09g006390.1.t1	343	37992.78	5.77	48.9	77.99	-0.51
*DlMYB65*	Dil.09g006410.1.t1	342	38051.05	7.13	42.67	75.67	-0.523
*DlMYB66*	Dil.09g006470.1.t1	343	37992.78	5.77	48.9	77.99	-0.51
*DlMYB67*	Dil.09g006490.1.t1	342	38051.05	7.13	42.67	75.67	-0.523
*DlMYB68*	Dil.09g009110.1.t1	352	39806.56	6.41	56.12	67.05	-0.77
*DlMYB69*	Dil.09g010800.1.t1	312	34140.01	8.32	57.27	68.49	-0.654
*DlMYB70*	Dil.09g012320.1.t1	189	21611.01	10.27	63.2	53.7	-1.026
*DlMYB71*	Dil.09g016170.1.t1	339	38332.43	9.16	54.27	55.58	-0.9
*DlMYB72*	Dil.09g019990.1.t1	285	32208.29	8.84	53.73	69.19	-0.732
*DlMYB73*	Dil.10g009380.1.t1	235	26851.36	9.06	56.53	72.68	-0.967
*DlMYB74*	Dil.10g011020.1.t1	326	37131.26	5.87	53.77	64.02	-0.756
*DlMYB75*	Dil.10g012030.1.t1	359	39992.12	6.52	48.07	69.61	-0.605
*DlMYB76*	Dil.10g014520.1.t1	500	55686.56	6.02	54.28	50.78	-0.714
*DlMYB77*	Dil.10g015060.1.t1	516	58504.42	8.83	59.13	69.19	-0.88
*DlMYB78*	Dil.10g016910.1.t1	207	23787.97	7.14	45.48	80.48	-0.671
*DlMYB79*	Dil.10g021960.1.t1	367	41320.98	6.04	57.84	66.13	-0.684
*DlMYB80*	Dil.11g017840.1.t1	255	28127.33	8.53	44.77	67.02	-0.791
*DlMYB81*	Dil.11g021760.1.t1	292	32450.63	9.02	48.16	66.85	-0.716
*DlMYB82*	Dil.12g003640.1.t1	530	57302.29	5.98	56.51	61.74	-0.687
*DlMYB83*	Dil.12g005130.1.t1	277	31223.66	5.72	54.12	64.84	-0.798
*DlMYB84*	Dil.12g005140.1.t1	254	29246.63	5.84	55.57	62.24	-0.885
*DlMYB85*	Dil.12g005190.1.t1	351	39488.21	5.62	47.99	84.16	-0.522
*DlMYB86*	Dil.12g009920.1.t1	285	33122.79	5.25	45.41	69.05	-0.975
*DlMYB87*	Dil.12g011130.1.t1	417	47372.34	5.93	43.57	65.06	-0.895
*DlMYB88*	Dil.12g011140.1.t1	427	50027.22	9.81	58.11	76.93	-0.805
*DlMYB89*	Dil.12g011220.1.t1	188	21745.41	6.43	64.88	63.88	-0.863
*DlMYB90*	Dil.12g011380.1.t1	206	23654.98	8.94	50.83	78.5	-0.648
*DlMYB91*	Dil.12g012160.1.t1	331	36962.38	6.06	37.44	78.97	-0.592
*DlMYB92*	Dil.12g022300.1.t1	192	21652.57	9.39	53.43	75.68	-0.724
*DlMYB93*	Dil.13g001590.1.t1	423	48422.9	8.85	48.26	64.54	-0.945
*DlMYB94*	Dil.13g001920.1.t1	308	34595.9	9.27	52.13	61.43	-0.722
*DlMYB95*	Dil.13g007520.1.t2	364	40814.16	7.02	51.44	51.21	-0.81
*DlMYB96*	Dil.13g010640.1.t1	341	37820.08	7.15	48.78	70.91	-0.742
*DlMYB97*	Dil.13g014250.1.t1	340	37954.54	6.42	66.15	62.79	-0.674
*DlMYB98*	Dil.13g016120.1.t1	140	16197.51	10.01	68.5	68.93	-0.952
*DlMYB99*	Dil.14g001000.1.t1	367	40730.4	6.04	54.9	67.47	-0.608
*DlMYB100*	Dil.14g003650.1.t1	281	31773.69	8.51	49.05	56.23	-0.766
*DlMYB101*	Dil.14g004030.1.t1	396	42941.23	6.77	39.59	66.01	-0.54
*DlMYB102*	Dil.14g005230.1.t1	353	40881.79	9.5	59.48	66.94	-0.943
*DlMYB103*	Dil.14g008020.1.t1	339	37657.01	8.25	51.53	57.55	-0.676
*DlMYB104*	Dil.14g008230.1.t1	140	16404.98	9.97	62.9	75.93	-0.846
*DlMYB105*	Dil.14g014450.1.t1	382	43716.84	8.9	65.51	74.82	-0.805
*DlMYB106*	Dil.14g015070.1.t1	250	29092.65	9.03	58.68	63.96	-0.964
*DlMYB107*	Dil.14g017610.1.t1	414	45261	6.64	52.4	64.61	-0.748
*DlMYB108*	Dil.14g017800.1.t1	474	53058.4	6.57	52.74	53.16	-0.784
*DlMYB109*	Dil.14g018400.1.t1	339	37898.04	6.42	59.9	63.69	-0.83
*DlMYB110*	Dil.15g002360.1.t1	253	28917.6	9.2	54.88	72.09	-0.838
*DlMYB111*	Dil.15g002370.1.t1	260	30468.4	8.42	44.31	75.38	-0.937
*DlMYB112*	Dil.15g003650.1.t1	141	16379.59	9.68	55.65	65.67	-0.917
*DlMYB113*	Dil.15g005450.1.t1	382	42489.3	5.7	51.35	65.39	-0.66
*DlMYB114*	Dil.15g006420.1.t1	220	25476.55	7.01	35.62	70.86	-0.827
*DlMYB115*	Dil.15g006590.1.t1	287	33123.14	8.88	50.91	64.56	-0.966
*DlMYB116*	Dil.15g007070.1.t1	369	41802.42	5.72	52.21	59.73	-0.663
*DlMYB117*	Dil.15g009820.1.t1	202	23716.88	8.34	57.84	79.65	-0.8
*DlMYB118*	Dil.15g010740.1.t1	271	30202.85	5.05	53.07	75.28	-0.633
*DlMYB119*	Dil.15g013880.1.t1	195	21873.77	8.87	36.95	75.08	-0.777
*DlMYB120*	Dil.15g015010.1.t1	297	34410.41	6.18	61.08	62.69	-0.773
*DlMYB121*	Dil.15g015540.1.t1	295	31645.2	8.08	54.08	63.22	-0.598
*DlMYB122*	Dil.15g018030.1.t1	328	36663.67	6.4	49.63	65.12	-0.78
*DlMYB123*	Dil.15g018520.1.t1	249	28992.44	9.14	56.92	63.9	-0.929
*DlMYB124*	Dil.15g018960.1.t1	360	40514.42	5.74	53.73	69.92	-0.59

### Phylogenetic analysis of longan R2R3-MYB gene family members

**Fig. 1:**
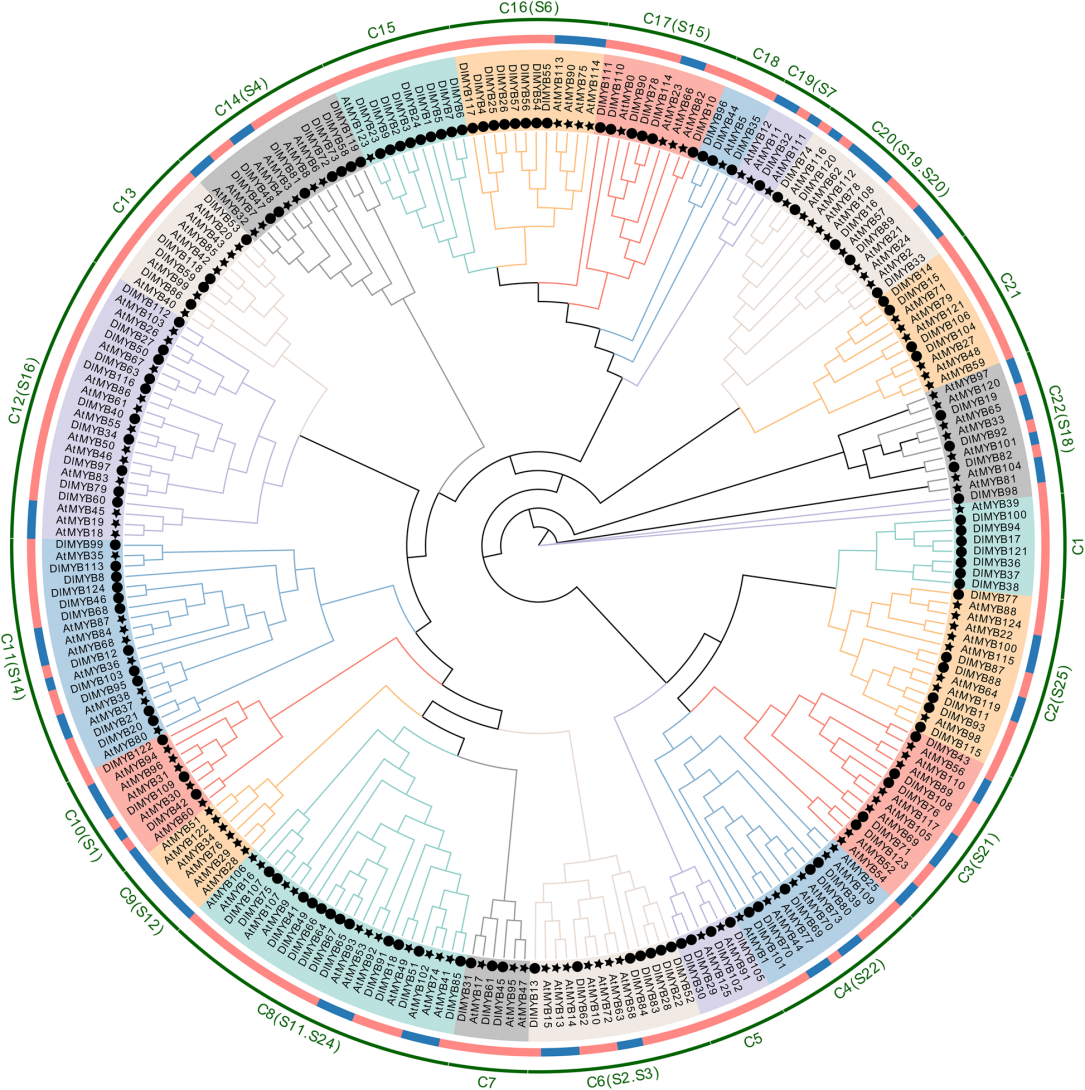
Phylogenetic tree of R2R3-MYB genes in *Dimocarpus longan* and *Arabidopsis thaliana*. The inner ring black star and black circle represent the members of R2R3-MYB gene family in *Arabidopsis* and longan, respectively. The middle blue leaf label represents the known *Arabidopsis* subgroup members, and the pink leaf label represents the longan subgroup members. The outer circle is represented as different subfamilies, C1 ~ C22 are the names of different subgroups of longan R2R3-MYB, and the S group in brackets is the known *Arabidopsis* subfamily name, this figure was created by the first author, Xinmin Lv

A phylogenetic tree was constructed using the conserved amino acid sequences of R2R3-MYB gene family members from both Longan and *Arabidopsis*. The 124 members of the R2R3-MYB gene family in *Arabidopsis* were classified into 22 subfamilies, denoted by C1 to C22 (Fig. 1) [4]. Eighteen of the R2R3-MYB gene subfamilies in *Arabidopsis* contained some known *Arabidopsis* subfamilies whereas six were inconsistent with the reported classification results of *Arabidopsis* subfamilies. The C9 subfamily, unique to *Arabidopsis,* did not contain any longan R2R3-MYB genes among its members. All subfamilies had more than six members, and the largest number of members was the C8 subfamily, with 24 family members. The phylogenetic analysis results suggest a potential correlation between the subfamily classification of longan R2R3-MYB genes and their functional similarities.

### Analysis of gene structures, conserved motifs and domains of longan R2R3-MYB gene family members

**Fig. 2:**
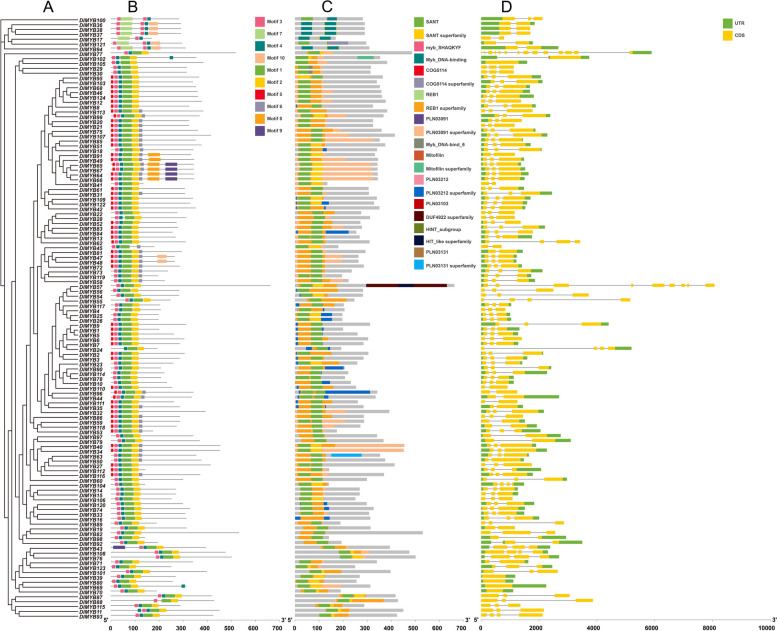
Gene structures of R2R3-MYB genes in *Dimocarpus longan*. **A** phylogenetic analysis; **B** conserved motif analysis; **C** protein conserved domain analysis; **D** gene structure display

The analysis of 124 longan R2R3-MYB members included the examination of conserved motifs, conserved domains, and gene structures. The results of conserved motif analysis showed that all R2R3-MYB family members contained four highly conserved motifs; motif1, motif2, motif3, and motif5 (Fig. 2B). The positions of the conserved motifs were consistent with those of the conserved MYB domains, indicating that both the R2 and R3 domains of the R2R3-MYB proteins in longan are highly conserved (Fig. 2C). Additionally, it was observed that R2R3-MYB members within the same subfamily exhibited similar composition and distribution of motifs, as depicted in Fig. 2A. The conserved domain prediction results showed that all Longan R2R3-MYB members contained SANT and SNAT superfamily conserved domains (Fig. 2C). The number of introns of the longan R2R3-MYB gene family members ranged from 0 to 13, and the number of exons from 1 to 14. Moreover, a noteworthy observation was the substantial variation in the number of introns and exons among members belonging to distinct subfamilies. Conversely, members within the same subfamily exhibited greater similarity in terms of gene structure, as illustrated in Fig. 2D.

### Chromosomal distribution and colinearity analysis of R2R3-MYB gene family members in longan

**Fig. 3:**
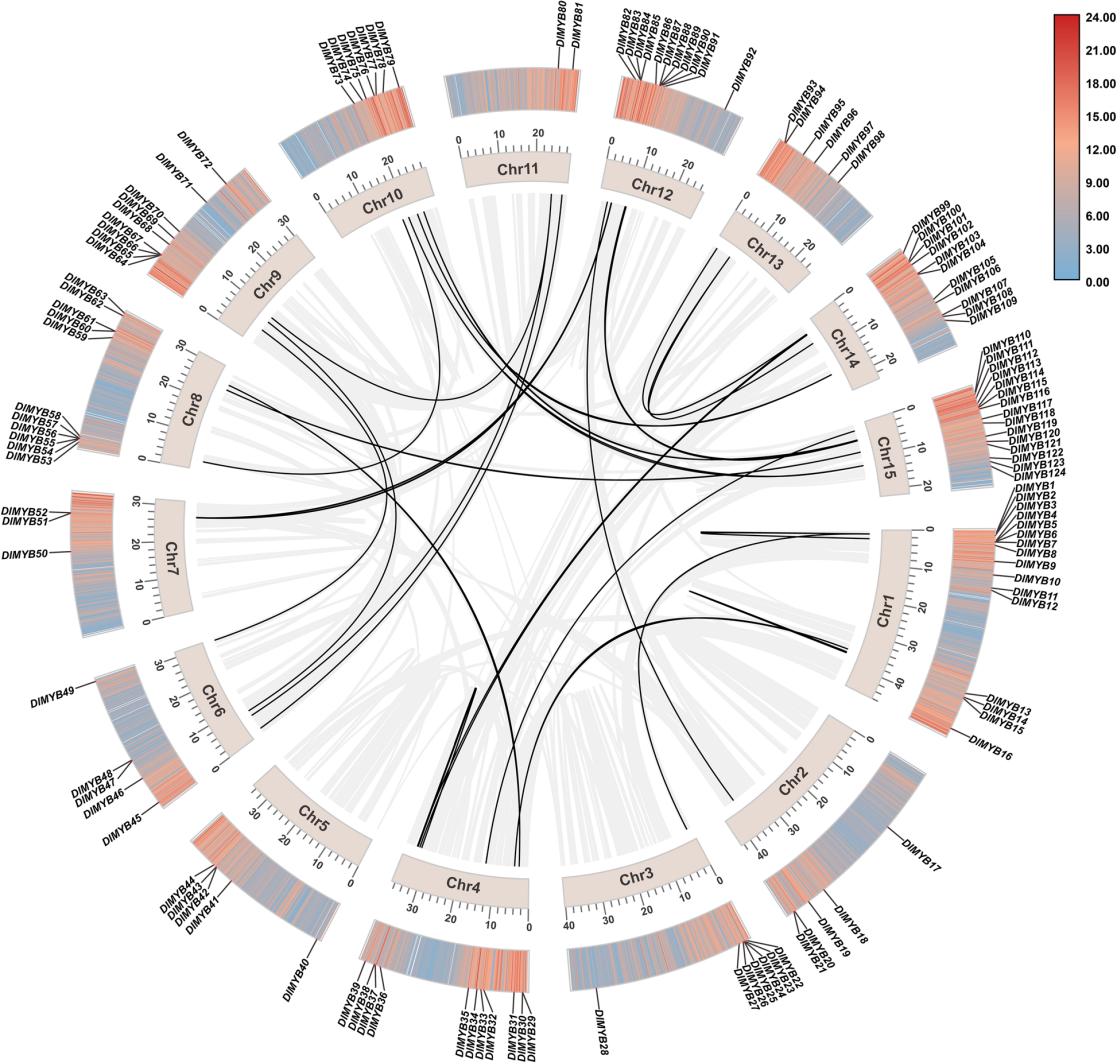
Chromosomal location and collinearity analysis of longan R2R3-MYB family genes. Jasmine boxes represent chromosomes. Segmental duplication genes are connected with black lines

We visualized the chromosomal distribution and colinearity of the R2R3-MYB gene family members in longan. The results showed that the members were unevenly distributed on the 15 longan chromosomes (Fig. 3). The largest number of R2R3-MYB gene family members, comprising of 15, was found on Chr15 and the smallest number, comprising of 2, was on Chr11 (Fig. 3). This indicates that the number of R2R3-MYB gene family members distributed on each chromosome did not correlate with the length of the chromosome. The colinearity analysis showed several genes duplicated in tandem among the members of the R2R3-MYB gene family in longan, such as DlMYB1, DlMYB2, DlMYB3, DlMYB5, and DlMYB6, in addition to 15 pairs of fragmented duplicated genes (Fig. 3). The above results suggest that longan R2R3-MYB genes are mainly generated by chromosomal fragment duplication, which is distant on the chromosome. An unequal interchange of alleles generates a small number of tandem duplicated genes, resulting in homologous sequence clusters.

### Tissue expression analysis of R2R3-MYB gene family members in longan

**Fig. 4:**
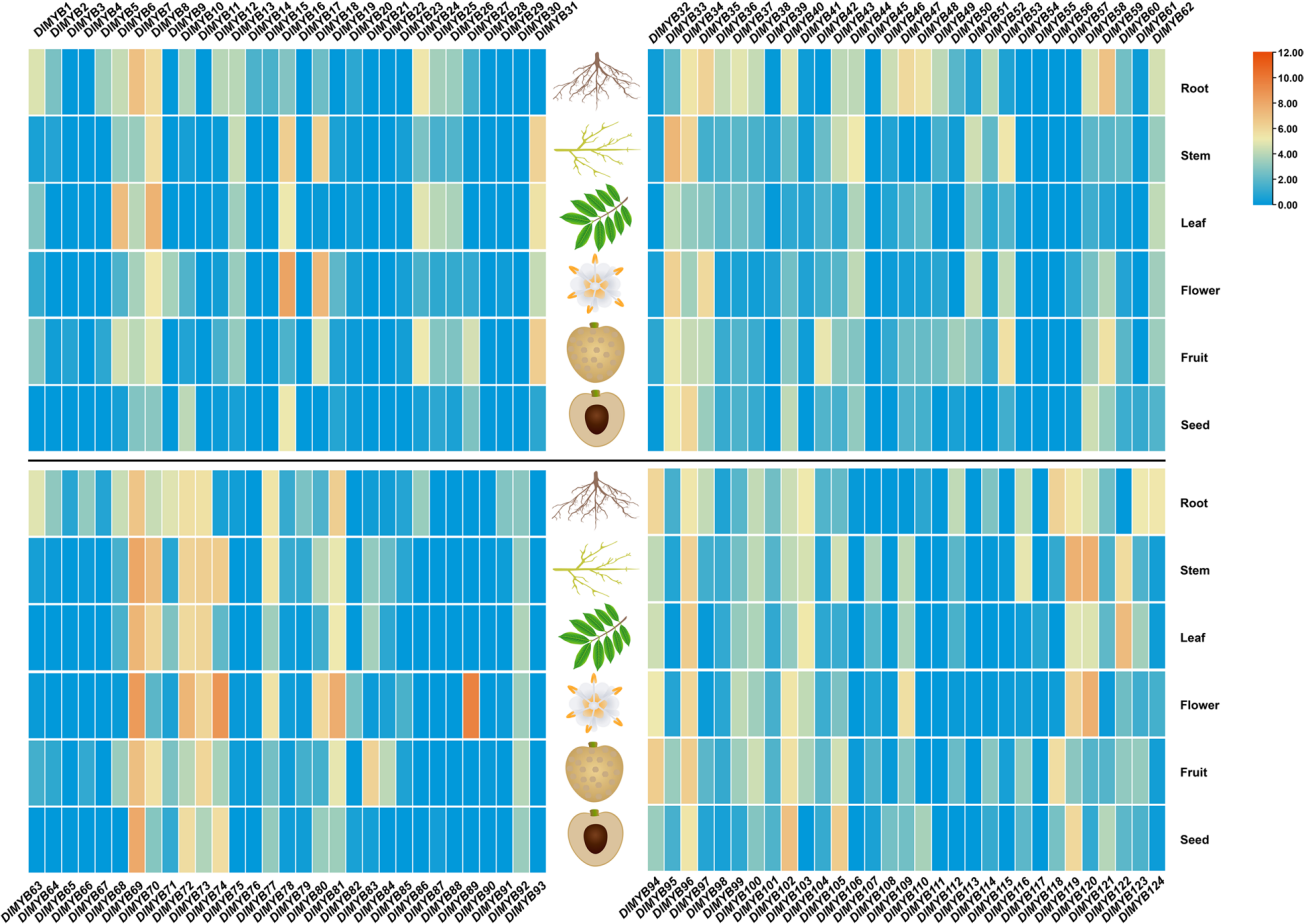
The expression of R2R3-MYB gene in different tissues of longan. The expression level differences of the longan R2R3-MYB gene family members in the roots, stems, leaves, flowers, fruits, and seeds of longan are shown

Multiple Experiment Comparison types of Gene Expression analysis were conducted using SapBase (Sapindaceae Genomic DataBase) (http://www.sapindaceae.com/index.html). The obtained expression values of certain longan R2R3-MYB gene family members encompassed various plant tissues, including roots, stems, leaves, flowers, fruits, and seeds. The results showed that eight members, including DlMYB69, DlMYB72, and DlMYB74, were the most highly expressed in longan flowers (Fig. 4). Thirteen members, including DlMYB6, were the most highly expressed in leaves. Thirteen members, including DlMYB7, were the most highly expressed in roots. Fourteen members, including DlMYB33, were the most highly expressed in stems. Sixteen members, including DlMYB69, were the most highly expressed in fruits. DlMYB69 had the highest expression in all tissues.

**Fig. 5:**
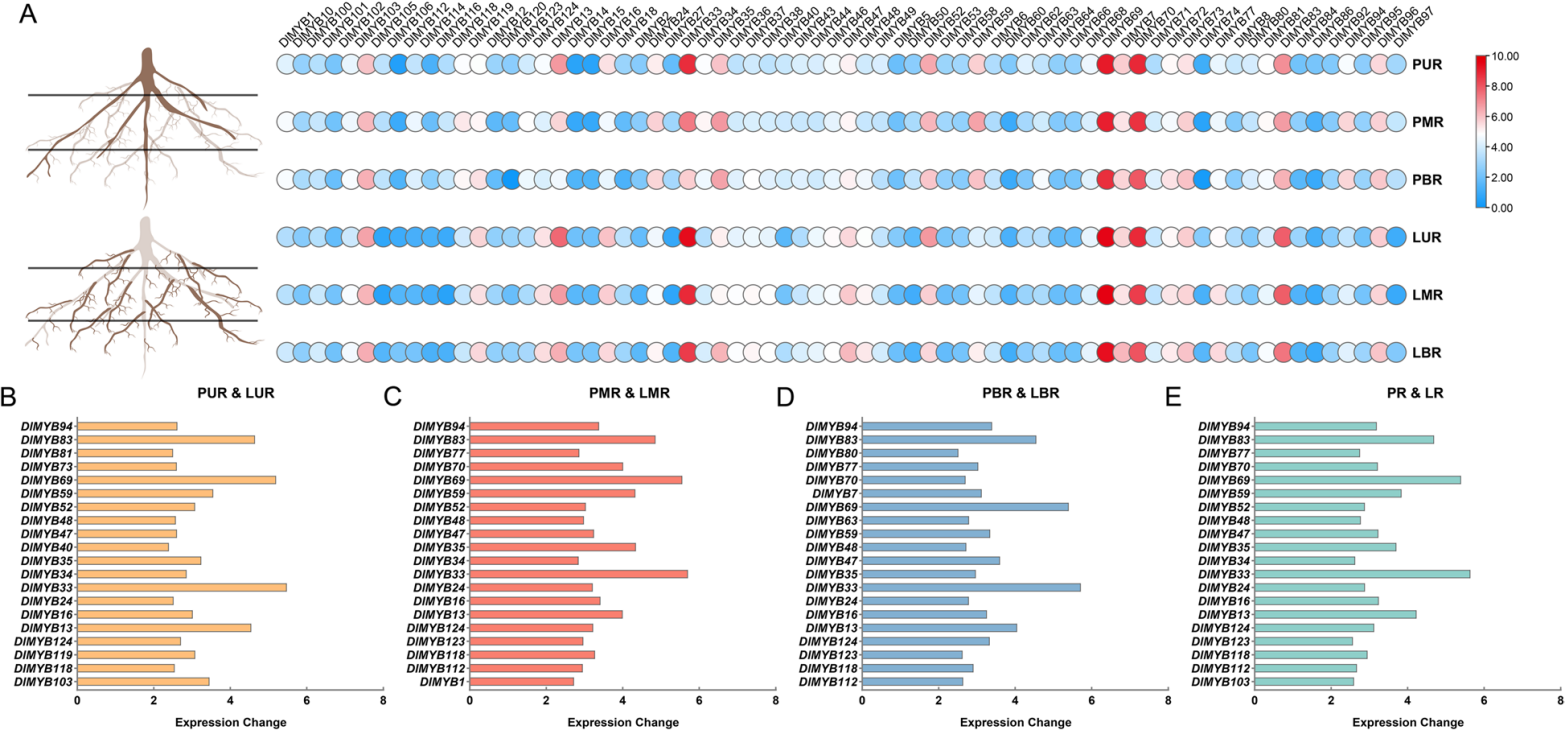
The expression of R2R3-MYB gene in primary root and lateral root of longan. **A** Heat map of R2R3-MYB gene expression in primary roots and measured roots of longan; the bar chart of the top 20 R2R3-MYB genes with the largest difference in expression between the primary root and the upper **B** middle **C**, lower **D** and overall **E** root of longan

In order to gain a deeper understanding of the potential role of R2R3-MYB transcription factors (TFs) in the development of longan roots, an analysis was conducted to examine the expression of R2R3-MYB genes in both primary and lateral roots of longan. This analysis utilized root RNA-seq data from the PRJNA554213 dataset. As shown in Fig. 5A, DlMYB33 and DlMYB83 were more expressed in lateral roots than in primary roots, while DlMYB69 and DlMYB70 had the highest expression in both primary and lateral roots. To screen for R2R3-MYB that may regulate lateral root development in longan, we further differentially showed the top 20 MYB TFs with the greatest variation in the expression on the top (Fig. 5B), middle (Fig. 5C), bottom (Fig. 5D), and overall (Fig. 5E) of the main and lateral roots. The results showed that the largest difference in expression was in DlMYB33, followed by DlMYB69, DlMYB83, and DlMYB13.

### Prediction of miRNAs regulating R2R3-MYB in longan

In order to investigate the potential regulatory role of miRNAs on R2R3-MYB gene expression in longan, miRNA prediction was carried out using the SapBase website and miRNA sequencing data. A total of 34 miRNAs were predicted, which are likely to exert post-transcriptional regulatory effects on R2R3-MYB genes in longan. The details of these predicted miRNAs are provided in Table 2. Among all predicted miRNAs, the number of novel miRNAs was 17. Different miRNAs were found to regulate the same R2R3-MYB, and majorly DlMYB98, DlMYB92, DlMYB103, and DlMYB115 (background markers in different colors in Table 2). The corresponding homologous genes of these R2R3-MYB family members in *Arabidopsis* were determined through sequence comparison and analysis. The homologs of DlMYB98 and DlMYB92 were found to be GAMYB and AtMYB33, respectively, two MYB TFs reported to be closely associated with root development in *Arabidopsis* [15].

**Table 2:**
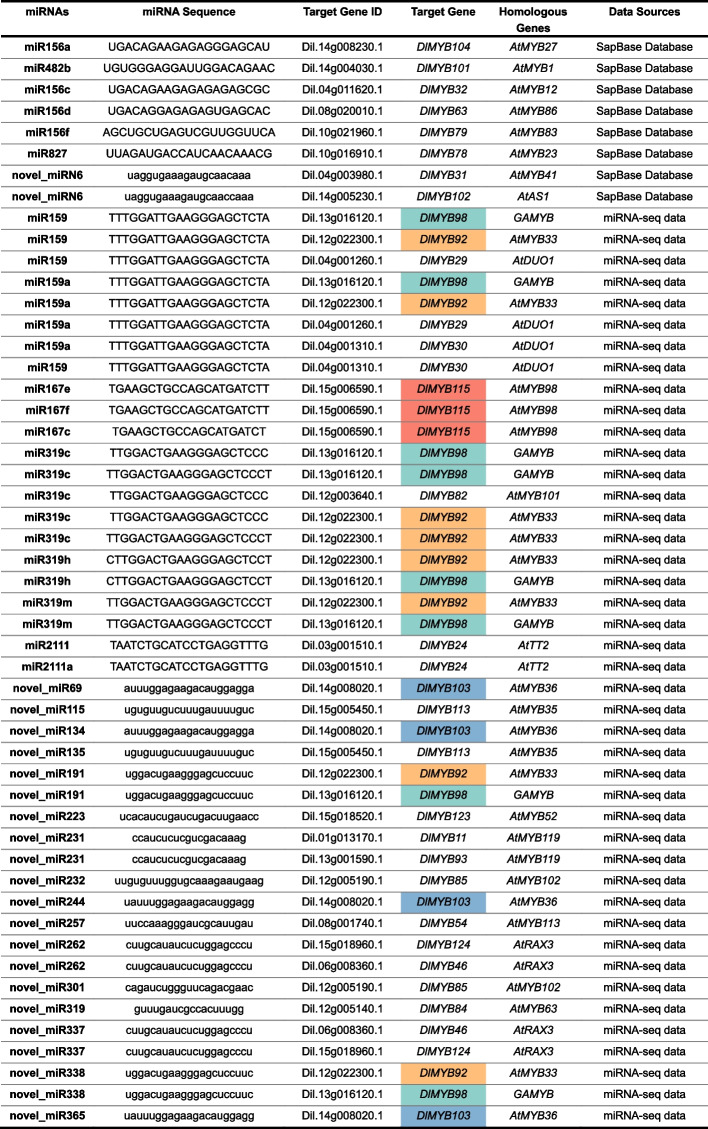
Prediction of miRNAs regulating longan R2R3-MYB gene

The uppercase letters represent the mature miRNA sequences known in longan, while the lowercase letters represent the mature miRNA sequences predicted in longan. The four different background colors in the table highlight the *DlMYB* family, which has the highest number of members in longan that can be cleaved by miRNAs

### qRT-PCR analysis of differential R2R3-MYB gene expression in longan primary and lateral roots

**Fig. 6:**
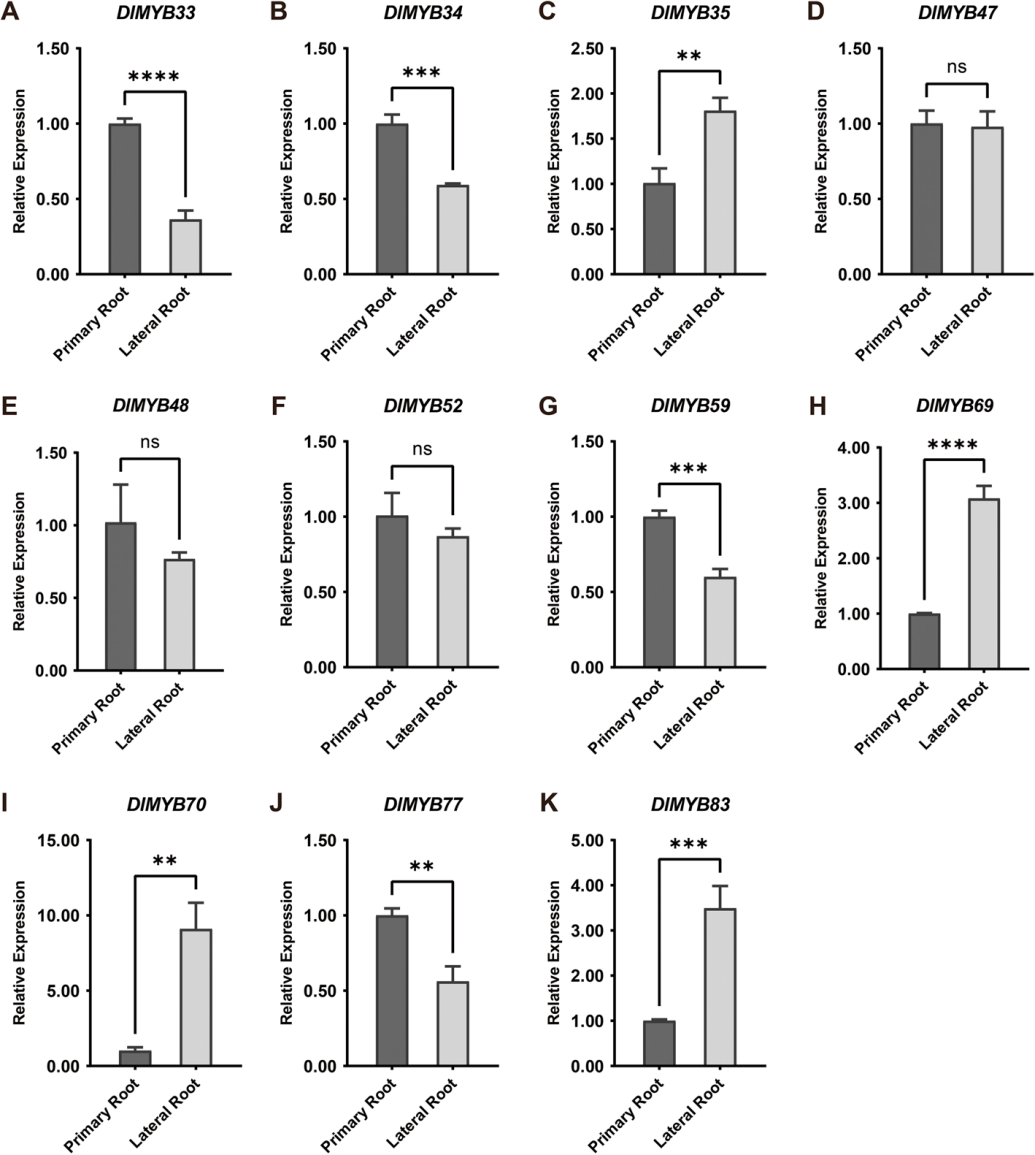
Analysis of relative expression of 11 R2R3-MYB genes from *Dimocarpus longan*. Relative expression of **A** *DlMYB33*, **B** *DlMYB34*, **C** *DlMYB35*, **D** *DlMYB47*, **E** *DlMYB48*, **F** *DlMYB52*, **G** *DlMYB59*, **H** *DlMYB69*, **I** *DlMYB70*, **J** *DlMYB77* and **K** *DlMYB83* in longan primary and lateral root

The expression of eleven R2R3-MYBs in the primary and lateral roots was randomly detected using qRT-PCR, as shown in Fig. 5E. It was observed that *DlMYB33*, *DlMYB34*, *DlMYB59*, and *DlMYB77* exhibited significant upregulation in the primary roots compared to the lateral roots of longan (Fig. 6). Additionally, *DlMYB45* and *DlMYB52* showed higher expression in the primary roots than in the lateral roots, although the differences were not statistically significant. *DlMYB35*, *DlMYB69*, *DlMYB70* and *DlMYB83* were less expressed in primary roots, i.e., the present residence, than in lateral roots. Inevitably, variations in the expression levels of the same genes were observed between qRT-PCR and RNA-seq results due to inconsistencies in the test materials and normalization methods employed (Fig. 6).

### Analysis of subcellular localization and expression analysis of DlMYB92

**Fig. 7:**
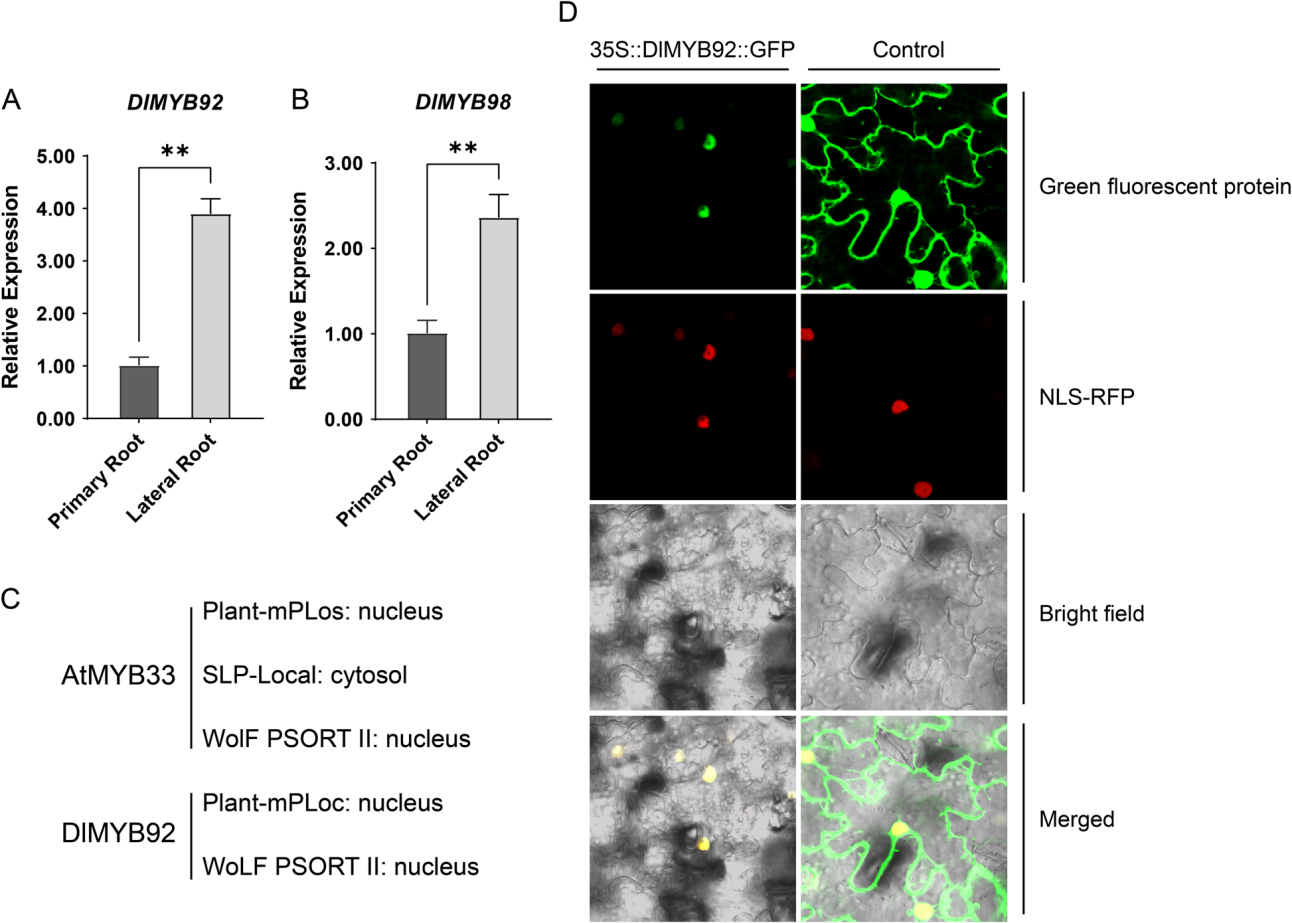
Expression and subcellular localization analysis of DlMYB92 and DlMYB98. Expression analysis of DlMYB92 (**A**) and DlMYB98 (**B**) in root and lateral roots of longan. **C** Prediction of subcellular localization of DlMYB92 and DlMYB98 in longan. **D** Subcellular localization of DlMYB92 in tobacco leaves

MYB33 and AtGAMYB are widely studied TFs involved in plant root development in *Arabidopsis*. Our results showed that in longan, DlMYB92 (AtMYB33 homolog) and DlMYB98 (AtGAMYB homolog) were regulated by multiple miRNAs and differed significantly in expression in primary and lateral roots. The expression of DlMYB92 and DlMYB98 in the primary and lateral roots of longan was further examined using qRT-PCR. The results showed that the expression of DlMYB92 and DlMYB98 in the primary roots was significantly lower than that in the lateral roots, consistent with the results in Fig. 7A, B. The subcellular localization of DlMYB92 and AtMYB33 was also predicted. As shown in Fig. 7C, most predictions showed that both DlMYB92 and its homolog in *Arabidopsis*, AtMYB33, were localized in the nucleus. Next, we performed a subcellular localization assay for DlMYB33 in tobacco leaves. The results showed that DlMYB92 was localized in the nucleus of tobacco cells compared with the negative control and NLS-nuclear localization signal (Fig. 7D). It is speculated that *DlMYB33* plays a transcriptional regulatory role in the nucleus and may be involved in the regulation of longan lateral root development.

## Discussion

### Longan R2R3-MYB family members display evolutionary conservation along with interspecies diversity

In this study, we identified a total of 124 members belonging to the R2R3-MYB transcription factor family in longan. By comparing the number of R2R3-MYB genes in longan with other species such as jujube [27], pear [28], *Arabidopsis* [3], soybean [29], and pea [30], we observed quantitative variation in R2R3-MYB gene numbers among different species. This variability can be attributed to differences in the occurrence of whole genome duplication (WGD) events in plants across species. Some species may have a lower number of R2R3-MYB genes due to the loss of genes that expanded after the WGD event. Phylogenetic analysis of R2R3-MYB family members in longan and *Arabidopsis* revealed that 18 out of the 22 longan R2R3-MYB gene subfamilies matched with known *Arabidopsis* subfamilies. However, six subfamilies did not align with the reported classification of *Arabidopsis* subfamilies [3]. These findings suggest that functional adaptive differentiation of R2R3-MYB genes may have occurred in longan and *Arabidopsis* following the WGD event. Furthermore, our phylogenetic and genetic structure analyses demonstrated that members of the same R2R3-MYB subfamily in longan share similar motifs and gene structures. This pattern is consistent with previous studies conducted in other species, such as potato [31] and pepper [32]. In summary, although longan exhibits quantitative diversity in R2R3-MYB gene numbers compared to other plant species, these genes remain functionally conserved, indicating their evolutionary significance.

### Multiple miRNAs orchestrate the post-transcriptional regulation of Longan R2R3-MYB family members

miRNAs are a class of single-stranded endogenous non-coding RNAs that post-transcriptionally regulate gene expression [5]. They play an important role in plant growth and development, participating in developmental processes, signal transduction, protein degradation, response to adversity stress, and pathogen invasion [6]. Numerous studies have shown that miRNAs play a leading role in post-transcriptional regulation, especially in targeting transcription factors [10, 11]. MiRNAs that potentially regulate the R2R3-MYB members in longan were predicted using online prediction tools and miRNA sequencing. The results showed that miRNA abundance obtained from online database prediction was significantly less than that from miRNA sequencing. Therefore, a single online prediction approach may lead to the loss of the obtained miRNA abundance. Multiple miRNAs were found to have the potential to regulate the same R2R3-MYB gene, and a single miRNA was also found to have the potential to regulate different R2R3-MYB genes in longan (Supplemental Fig. 1). The longan R2R3-MYBs regulated by the largest number of miRNAs were DlMYB92, DlMYB98, DlMYB115 and DlMYB103. Among them, both DlMYB98 and DlMYB92 were cleaved by different members of the miR159 and miR319 families. The homologs of DlMYB92 and DlMYB98 in *Arabidopsis* were identified as AtMYB33 and AtGAMYB, respectively. These genes share a close evolutionary relationship in both longan and *Arabidopsis*, as reported by Dubos et al. [4]. This finding suggests that the same miRNA family can regulate members of the R2R3-MYB family with similar gene structures. It was observed that the R2R3-MYB genes regulated by the same miRNA did not show overlap in covariance results. This suggests that R2R3-MYBs regulated by the same miRNA are not functionally redundant and may exhibit differences in their regulatory roles.

### Conserved miRNA-MYB modules involved in root development are present in longan>

Out of the 124 identified R2R3-MYB family members in longan, 26 were found to be regulated by 34 miRNAs. Notably, 17 of these miRNAs were specific to longan and considered novel. The regulatory pathways governed by these miRNA-MYB interactions were directly associated with plant root development, highlighting their significance in this biological process. In *Arabidopsis*, miR159 has been shown to promote lateral root growth by targeting MYB transcription factors. Double mutants of *miR159ab* exhibit larger root tip meristem tissues, higher cell numbers, and longer primary roots compared to the wild type. miR159 negatively regulates the expression of *AtMYB33*, *AtMYB65*, and *AtMYB101* genes, leading to increased cell division and primary root growth in the root tip meristematic tissue [15]. In our study, we predicted the involvement of the miR159 gene family (including miR159a, miR159b, and miR159c) in the regulation of *DlMYB92* (homolog of *AtMYB33*) in longan.

Another regulator of longan R2R3-MYB is miR319. Previous research has demonstrated the existence of differentially expressed mRNAs and miRNAs during the development of radish (Raphanus sativus) tubers. Among them, miR319 candidate target genes, such as *RSG11844.t1*, *RSG42419.t1*, and *RSG49768.t1*, are involved in tuber formation and development [33]. In citrus, up-regulation of miR319 expression was observed in roots under 400 μmol/ L H_3_BO_3_ stress, resulting in the down-regulation of the target MYB gene and subsequent alteration of root morphological structure, including a decrease in root tip number [34]. We also discovered that *DlMYB98* exhibited significantly higher expression in lateral roots compared to primary roots and was regulated by miR319 in longan. In summary, our findings suggest the presence of a conserved mechanism involving miRNA-DlMYB regulation in root development in longan. However, further in-depth investigation is required to elucidate the precise mechanisms by which miR159 and miR319 regulate the expression of *DlMYB92* and *DlMYB98*, respectively, and subsequently influence longan root development.

## Methods

### Material

The materials used in this study were ‘Chu Liang’ longan, all from the Guangdong Longan Germplasm Resource Nursery. Longan seeds were sown in moist river sand in April 2023, and after about 30 days of growth, the resulting seedlings were used in the experiment. Primary and lateral root parts of the seedings were collected in triplicate. These samples were rapidly frozen in liquid nitrogen and stored in a -80℃ refrigerator for subsequent studies.

### Identification and physicochemical property analysis of the R2R3-MYB gene of longan

The longan genome and gene annotation files were obtained through the SapBase database (http://www.sapindaceae.com/index.html). HMM files were downloaded from the Pfam database (http://pfam-legacy.xfam.org/). A sequence search of the longan genome was performed using Simple HMM Search in TBtools software, using PF00249 as a model [35]. Candidate genes were then submitted to the Batch CDD-search (https://www.ncbi.nlm.nih.gov/Structure/cdd/wrpsb.cgi) database for comparison to verify the R2R3-MYB conserved domains and remove sequences without MYB domains or with incomplete domains. The isoelectric point, molecular mass, hydrophilic mean coefficient, instability coefficient, and aliphatic coefficient of the R2R3-MYB protein were calculated through Expasy online (https://www.expasy.org/).

### Phylogenetic characterization of longan R2R3-MYB family members

The ClustalW software compared the longan R2R3-MYB amino acid sequence with 124 *Arabidopsis* R2R3-MYB amino acid sequences. The Neighbor-Joining (NJ) method was used to construct a phylogenetic tree by MEGA software [36]. The members of the longan R2R3-MYB gene family were grouped with reference to the grouping of *Arabidopsis* in the phylogenetic tree [4].

### Analysis of conserved motifs, conserved domains, and gene structures of longan R2R3-MYB

Conserved motif analysis of the longan R2R3-MYB protein sequence was performed using the MEME online tool (https://meme-suite.org/meme/doc/meme.html), and the number of conserved motifs was set at 10. Conserved domain analysis of the longan R2R3-MYB protein sequence was performed using the CDD-search tool on the NCBI website. The exon, intron, and untranslated (UTR) information corresponding to the R2R3-MYB gene were extracted from the longan genome using TBtools software for gene structure analysis. Finally, the conserved motifs, conserved domains, and gene structures of the genes were synchronized with the phylogenetic tree of R2R3-MYB in longan using TBtools software for display.

### Chromosome distribution and colinearity analysis of R2R3-MYB gene in longan

Chromosomal location information of the R2R3-MYB genes was extracted from the longan genomic information using TBtools software and visualized for analysis. In addition, gene maps with duplicated fragments were selected and analyzed by the Multiple Colinearity Scanning Toolkit (MCSan X) for default parameters [37]. The synonymous relationship of the R2R3-MYB gene was determined by using Advanced Circos software in TBtools.

### qRT-PCR analysis

Total RNA was extracted from the primary and lateral roots of ‘Chu Liang’ longan using RNAprep Pure Polysaccharide Polyphenol Plant Total RNA Extraction Kit (DP441). cDNA was synthesized using PC44-THERMOscript 1st Strand cDNA Synthesis Kit (PC4402). mRNA was used as template to design *DlMYB33*, *DlMYB34*, *DlMYB35*, *DlMYB47*, *DlMYB48*, *DlMYB52*, *DlMYB59*, *DlMYB69*, *DlMYB70*, *DlMYB77*, *DlMYB83*, *DlMYB92* and *DlMYB98* quantitative primers (Supplemental Table 1). qRT-PCR analysis was performed using the QuantStudio 3 Real-Time PCR System. The reaction system was performed according to the instructions of the PC60-2 x SYBR Green qPCR Mix (Low ROX) kit, using DlActin as the internal reference gene. Each sample run was repeated thrice, and the relative gene expression was calculated using the 2^−ΔΔCT^ method [38].

### Prediction of longan miRNA and miRNA sequencing analysis

The sRNA Target Prediction function in the online database SapBase was used to predict miRNAs capable of shearing the R2R3-MYB gene in longan. miRNA high-throughput sequencing was performed using ‘Chu Liang’ longan root as a sample, with three replicates set. miRNA library construction and high-throughput sequencing were co-performed with Biomarker Technologies Co and Beijing Biomarker Technologies Co The target gene analysis of miRNA was performed using BMKCloud (www.biocloud.net). The sequencing data are not yet available.

### Subcellular Localization of DlMYB92 in tobacco leaf cells

The full-length coding sequence (CDS) of *DlMYB92* was amplified by KOD FX using the cDNA of the longan root as a template. The target sequences were detected by gel electrophoresis and recovered, and the full-length CDS of *DlMYB92* was ligated to the linearized pRI101-AN vector using NEB Builder HIFI DNA Assembly. The recombinant plasmid was then transferred into Trans1-T1 Phage Resistant Chemically Competent Cell, and PCR was used to confirm presence of the recombinant vector.

The recombinant vector was transformed into GV3101(pSoup-p19) Chemically Competent Cell, and PCR identified the positive clones. The identified positive clone was incubated in LB medium containing rifampicin (50 mg/L) and kanamycin (50 mg/L) with shaking (28 °C, 200 r/ min) until the OD_600_ value of the bacterial solution was 1. The bacterial solution was collected by centrifugation at 4000 g for 10 min. The bacteria were suspended in an MS liquid medium with 10 mM MgCl_2_, 15 μM acetosyringone, and 10 mM MES, and the OD_600_ was adjusted to 0.6. Wild-type tobacco (*Nicotiana benthamiana*) in good growth condition was selected for Agrobacterium infiltration. Each leaf was injected with 0.5 mL of bacterial solution, and a non-recombinant vector was injected as a control. This was performed using an OLYMPUS FV3000 laser confocal microscope.

### Supplementary Information

**Additional file 1: Supplemental Figure 1.** Regulatory network of miRNAs and Longan MYB family members.

**Additional file 2: Supplemental Table 1.** Primers used in this study.

**Additional file 3.**

## Data Availability

The original contributions presented in the study are included in the article/Supplementary Material. Further inquiries can be directed to the corresponding author.
