# Supplementary material for: Genome-wide identification of the longan R2R3-MYB gene family and its role in primary and lateral root
Source: BMC Plant Biol. 2023 Sep 23;23:448. doi: 10.1186/s12870-023-04464-9 (PMC10517564; doi:10.1186/s12870-023-04464-9)
Supplement: Supplementary file 1 — Additional file 1: Supplemental Figure 1. Regulatory network of miRNAs and Longan MYB family members. [file 12870_2023_4464_MOESM1_ESM.pdf]

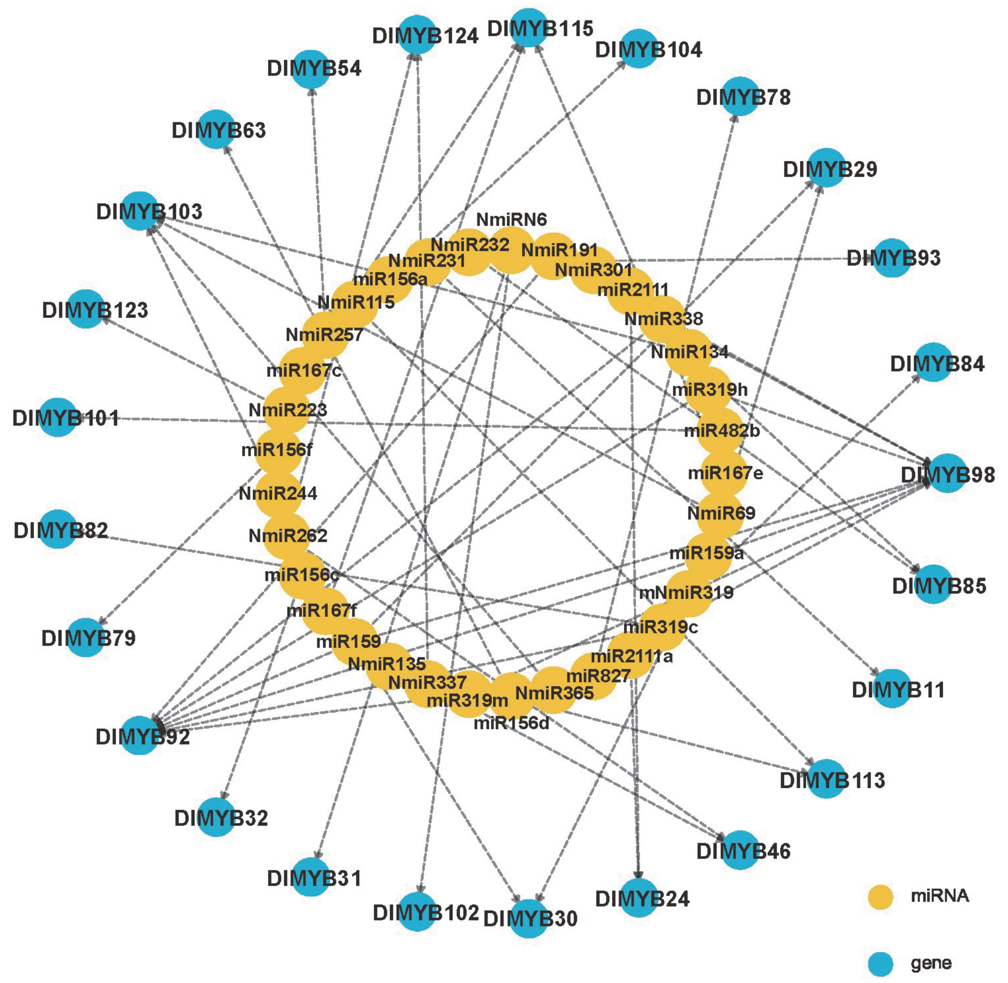

Supplemental Figure 1. Regulatory network of miRNAs and Longan MYB family members. The cleavage relationship between miRNAs and members of the longan R2R3-MYB transcription factor family is illustrated in the diagram. Yellow solid circles represent candidate miRNAs that may cleave the longan R2R3-MYB transcription factors, while blue solid circles represent longan R2R3-MYB genes that may be cleaved by miRNAs. this figure was created by the first author, Xinmin Lv.
